# Growth and economic performance of Kacang goats fed concentrates containing cattle rumen content silage

**DOI:** 10.5455/javar.2025.l996

**Published:** 2025-12-25

**Authors:** Edwin Jermias Lodowik Lazarus, Benny Yohanes Wole, Maritje Aleonor Hilakore, Emma Dyelim Wie Lawa

**Affiliations:** Animal Husbandry Study Program, Faculty of Animal Husbandry, Marine Science and Fisheries, Nusa Cendana University, Jl. Adisucipto, Penfui, P.O. Box, Kupang, East Nusa Tenggara, Indonesia

**Keywords:** Concentrate, economic efficiency, growth performance, Kacang goats

## Abstract

**Objective:**

This study aimed to determine the growth and economic efficiency of Kacang goats that were fed different levels of cattle rumen content silage (CRCS) in a concentrate ration based on Andropogon timorensis grass.

**Materials and Methods:**

Twenty male Kacang goats aged 8–10 months, with an average live weight of 11.7 ± 1.4 kg, were used as experimental animals. The treatment applied was the use of CRCS in the concentrate at different levels: 0% (T0), 10% (T1), 20% (T2), and 30% (T3). The feed consisted of 70% A. timorensis grass and 30% concentrate. Each treatment was repeated five times, resulting in 20 experimental units. The parameters measured were dry matter intake (DMI), daily weight gain (DWG), feed conversion ratio (FCR), feed cost per gain (FCPG), and income over feed cost (IOFC). The data were analyzed using analysis of variance and further tested with Duncan’s multiple range test.

**Results:**

The results showed that the treatment had no significant effect (*p* > 0.05) on DMI, while DWG, FCR, FCPG, and IOFC were significantly affected (*p* < 0.05) by the treatment. The use of 10% CRCS (T1) resulted in significantly higher (*p* < 0.05) DWG (38.20 gm/day), FCR (8.33), FCPG (40.57), and IOFC (Indonesian rupiahs 30,001) compared to T2 and T3, but was not significantly different (*p* > 0.05) from T0.

**Conclusions:**

The use of 10% CRCS in concentration improves the growth and economic efficiency of Kacang goats and provides sustainable benefits to the environment by utilizing CRCS in feed rations.

## Introduction

Ruminant livestock, including goats in tropical and subtropical regions, depend largely on the availability and quality of feed for their growth and productivity. The consistency of feed supply and quality fluctuates according to the season [1]. This condition poses a significant challenge to goat productivity, as feed costs are the largest component in a livestock business and its sustainability [2,3].

The intermittent availability of feed has led farmers to rely on agricultural waste, which is generally of low quality, as the main source of feed [4]. In this situation, the use of concentrates in rations is a strategy to improve the efficiency and performance of livestock consuming low-quality feed [5–7]. In various studies, the use of concentrated feed in ruminant feed rations has been shown to improve growth [8]. However, high prices and limited availability remain obstacles for most farmers in tropical regions [9].

The search for alternative feed concentrates is a strategic step in overcoming the scarcity and high prices of existing feed concentrates. The use of feed ingredients derived from agro-industrial waste and slaughterhouse waste has the potential to reduce feed costs and improve the sustainability of livestock farming systems [10]. To improve efficiency and competitiveness in small livestock businesses, these efforts need to be accelerated [11].

One waste that has potential as an alternative feed ingredient is cattle rumen contents, which is the digestive residue from abattoirs. Rumen content is a potential novel feed ingredient comprising digested feedstuffs at various stages of degradation, saliva, microorganisms, and fermentation products [12,13]. The rumen content, as well as cattle feces, still consists of high organic matter (OM) content [14]. Nevertheless, efforts are being made to increase the value of rumen content obtained from slaughterhouses, thereby reducing both production costs in the livestock industry and the environmental pollution it generates [15,16]. The method used to process rumen contents can be done through substrate enrichment (fortification) and fermentation processes [17].

The volume of this waste is also quite large. Kocu et al. [18] reported that rumen contents can reach 8%–10% of the live weight of cattle before slaughter. If an abattoir slaughters 20 cattle with an average weight of 200 kg/day, it can produce about 320–400 kg of rumen contents every day. However, direct use of rumen contents as feed faces challenges due to their low palatability and high moisture content. Although the high moisture content and unpleasant smell of rumen content are the primary obstacles, the use of rumen content with proper processing can provide a valuable source of nutrients when used as a supplement in the diets of various livestock species [19].

Therefore, further processing is required to improve its quality, one of which is through the ensilage process. Ram et al. [20] reported that fermentation of rumen contents into silage can improve the physical and chemical properties of the material, making it more suitable for consumption by livestock.

Several previous studies have assessed the use of dry rumen content in small livestock. Mondal et al. [21] tested the use of 0%, 5%, and 10% dry rumen content in goats, while Olafadehan et al. [22] evaluated the administration of 0%–60% in sheep. Osman et al. [23] added dry rumen content (0%, 5%, and 10%) to sheep concentrate rations, and Al-Wazeer [24] concluded that replacing part of the barley grain and soybean meal with 10% dry rumen content resulted in the best performance.

However, empirical evidence regarding safe and optimal addition levels remains limited, and results vary across studies. Furthermore, almost all studies use dry rumen content, while studies on rumen content of cattle that have been processed through ensilage [cattle rumen content silage (CRCS)] are still very rare. Therefore, this study was conducted to fill this gap by evaluating the use of CRCS in Kacang Goat concentrate.

To our knowledge, this is the first study to evaluate graded levels of CRCS in concentrate diets for indigenous Kacang goats, simultaneously assessing growth performance and economic efficiency. This work provides new insights into the practical use of abattoir waste as a low-cost, sustainable feed ingredient in smallholder goat production systems.

## Materials and Methods

### Ethical approval

The research protocol for this study was approved by the Ethics Committee of the Faculty of Animal Husbandry, Marine Science, and Fisheries, Nusa Cendana University (Approval Number: No/Ref: 131/1.KT/KEPPKP/IX/2024).

### Livestock and husbandry management

This study used 20 growing Kacang-type male goats, aged 8–10 months, with an average initial body weight (BW) of 11.713 ± 1.431 kg. The animals were kept for 70 days in individual cages measuring 1.5 × 0.7 m, equipped with feed and water containers. The goats were given a 1-week adaptation period to acclimate them to the research conditions, particularly the application of feed treatment. Before treatment, goats were given anthelmintic (Fenbendazole, 5 mg/kg BW) orally and underwent an adaptation period of 1 week.

The ration consisted of 70% kume grass (*And* *ropogon timorensi* *s*) and 30% concentrate, which was prepared to meet the crude protein (CP) requirement of 10.54% and total digestible nutrients (TDN) of 64% based on the recommendations of The National Research Council (NRC) [25]. Concentrate ingredients include rice bran, pollard, corn meal, fish meal, and CRCS.

### Preparation of CRCS

Cattle rumen content was obtained from the slaughterhouse in Kupang City, and then dried in the sun for 5 days until it reached a low moisture content (9.086%). Local microorganisms (LMO) were made by mixing cattle rumen fluid and coconut water in a 2:1 ratio. Silage materials consisted of cattle rumen contents, rice bran, and palm sugar, mixed with LMO as much as 4% of dry matter (DM) weight, from all mixtures of silage ingredients, then fermented for 4 weeks according to the method of Lina et al. [26]. After fermentation, the silage was dried for an additional 5 days until it was ready for use in the concentration formulation. Table 1 presents the nutrient composition of cattle rumen contents before and after the ensilage process.

**Table 1.** Nutrient content of cattle rumen contents and CRCS.

Nutrient (%)	Cattle rumen content	CRCS
DM	90.914	96.076
OM	86.113	89.107
CP	9.733	14.647
CF	23.983	19.284
Ether extract	5.829	10.524
Nitrogen-free extract	46.564	44.652
NDF	71.222	51.551
ADF	45.399	30.713
Gross energy (kcal/kg)	3941.24	4333.63

Analysis result of the Feed Chemistry Laboratory, Faculty of Animal Husbandry, Marine and Fisheries, Nusa Cendana University.

### Experimental design

The study used a with four treatments and five replications. Treatments consisted of the level of cattle rumen silage in concentrate, namely:

- T0 = 0% (without CRCS)

- T1 = 10%

- T2 = 20%

- T3 = 30%

Table 2 shows the composition of concentrating ingredients for each treatment, while Table 3 presents the nutrient content of the ration.

**Table 2.** Percentage of ingredients in the concentrate (%).

Feed ingredients	T0	T1	T2	T3
Rice bran	40	35	30	25
Pollard	30	27.5	25	22.5
Fine corn	25	22.5	20	17.5
Fish meal	5	5	5	5
CRCS	0	10	20	30
Total	100	100	100	100

**Table 3.** Nutrient content of the research ration.

Item	DM	OM	CP	CF	TDN
Kume grass	61.791	91.280	9.400	28.100	62.031
Concentrate	88.253	87.830	14.187	10.360	70.312
CRCS	96.076	89.107	14.647	19.284	61.470
T0	69.730	90.245	10.836	22.778	64.515
T1	69.964	90.283	10.850	23.046	64.250
T2	70.199	90.322	10.864	23.314	63.985
T3	70.434	90.360	10.878	23.581	63.720

### Measurement and research parameters

- DM intake (DMI)

Calculated by the formula:

DMI (gm/h/day) = DM fed − DM not consumed

- Daily weight gain (DWG)

Calculated by:

DWG (gm/head/day) = (final weight − initial weight)/maintenance duration

- Feed conversion ratio (FCR)

FCR = daily feed consumption (gm)/DWG (gm)

- Income over feed cost (IOFC)

IOFC describes the profit of the business after deducting feed costs.

IOFC (Rp/head/day) = (DWG × selling price/kg live weight) − daily feed cost

- Feed cost per gain (FCPG)

FCPG (Rp/kg gain weight) = daily feed cost/average daily gain (ADG)

### Sample collection and analysis

Forage and concentrates were fed and recorded daily, including feed residues, to calculate actual consumption. Feed given was taken once a week as a representative sample, while scraps were collected daily. Livestock were weighed at baseline and then on a weekly basis until the end of the period.

#### Laboratory analysis

Samples of feed and feed residue were analyzed for DM, OM, CP, and crude fiber (CF) content using the The Association of Official Analytical Chemists (AOAC) method [27].

#### Statistical analysis

The research data were tabulated and analyzed using analysis of variance to test the effect of treatment on the measured parameters. Differences between treatments were tested using Duncan’s multiple range test. The test was performed using SPSS software version 27.

## Results and Discussion

### Nutrient composition of the ration

Laboratory analysis data from cattle rumen contents and CRCS (Table 1) show changes in nutritional content. CP content increased by 66.45% from the initial material (9.733%) to 14.647%, while CF decreased from 23.983% to 19.284%, neutral detergent fiber (NDF) decreased significantly from 71.222% to 51.551%, and acid detergent fiber (ADF) from 45.399% to 30.713%. These findings align with the results reported by Lahay [28], thereby confirming that this silage becomes a higher-quality nutrient source (Table 1). The ration composition in the treatment (Table 3) shows that all formulations successfully met the CP requirement of ± 12% and TDN of ±64% according to NRC [25] recommendations for goats weighing 10–11 kg.

### Consumption and growth performance

According to Table 4, total DMI (grass + concentrate) did not differ significantly between treatments (*p* > 0.05). This means that adding up to 30% CRCS did not reduce intake, indicating that feed palatability was maintained. Although total intake tended to decrease at higher silage levels, this decrease was not significant (*p* > 0.05); this is likely due to slight changes in the physical and chemical quality of the diet with high silage content [29]. The dry intake of goat feed rations T0 and T1 was by Singh [30] recommendation for meat-type goats of 2.5%–3% of BW, and the NRC’s [25] recommendation for goats weighing 10–12 kg, which is 320–432 gm/h/day.

**Table 4.** Average dry matter intake, DWG, FCR, IOFC, and FCPG of Goats in the study.

Parameters	T0	T1	T2	T3	*p* -value
Dry matter intake					
Kume grass intake	240.21 ± 49.25	227.73 ± 20.32	187.30 ± 40.42	182.39 ± 29.59	0.05
Concentrate intake	95.67 ± 11.91	90.65 ± 11.75	98.19 ± 19.25	101.68 ± 17.20	0.72
Total intake (gm/h/day)	335.89 ± 57.35	318.37 ± 30.61	285.49 ± 51.63	284.06 ± 44.73	0.26
% BW	2.47	2.45	2.15	2.10	0.05
Initial weight (kg)	11.17 ± 1.16	10.73 ± 1.18	11.53 ± 1.83	11.92 ± 1.64	0.64
Final BW (kg)	13.60 ± 1.47	13.02 ± 1.34	13.29 ± 1.88	13.55 ± 1.71	0.90
Daily weight gain (gm/h/day)	40.52 ± 11.05ᵇ	38.20 ± 5.07ᵇ	29.41 ± 4.55ᵃ	27.20 ± 4.85ᵃ	0.02
FCR	8.29 ± 0.94ᵃ	8.33 ± 0.07ᵃ	9.06 ± 0.09ᵇ	10.45 ± 0.02ᵇ	0.05

Means on the same row with the same letter superscript are not statistically different (*p* < 0.05).

T0: Kume grass forage 70% + concentrate 30% (without CRCS)

T1: Kume grass forage 70% + concentrate 30% (concentrate contains 10% CRCS)

T2: Kume grass forage 70% + concentrate 30% (concentrate contains 20% CRCS)

T3: Kume grass forage 70% + concentrate 30% (concentrate contains 30% CRCS)

### Daily weight gain

DWG showed a significant difference (*p* < 0.05). Groups T0 and T1 had higher DWG (±40–38 gm/day), while T2 and T3 were relatively lower (29–27 gm/day). These results indicate that the inclusion of CRCS in the concentrate should not exceed 10% to achieve optimal growth. DWG in T1 was also higher than that of goats fed only *ad libitum* natural forage (approximately 27 gm/day), as reported by Adiwinarti et al. [31]. According to Yanti and Yayota [32], improving the nutritional content of feed is expected to increase livestock production.

The results of this study showed that the ADG of Kacang goats remained stable when CRCS was included up to 10% (≈38–40 gm/h/day) but declined significantly at higher inclusion levels (20%–30%). These findings are consistent with Al-Wazeer [24], who reported that total gain and ADG of Awassi lambs fed diets containing 10% and 20% dried rumen content (DRC) were not significantly different from those fed the control diet, although the 10% level tended to yield better performance. Similar results were also reported by Mondal et al. [21] and Osman et al. [23], who found that inclusion of DRC at 0%–10% in the diets of kids and lambs did not affect final BW or ADG. Meanwhile, Agolisi et al. [33] found that replacing up to 12% of soybean meals with DRC did not affect sheep performance.

### FCR

Feed conversion can be used to determine production efficiency because it is closely related to production costs. The lower the feed conversion value, the greater the efficiency of feed utilization. The FCR values at T2 and T3 were higher than those at T0 and T1, with a significant difference (*p* < 0.05), averaging 9.06–10.45 compared to 8.29–8.33 at T0 and T1. This indicates that feed utilization efficiency decreased at high silage levels. A higher FCR means more feed is required per unit of BW gain. This indicates low growth efficiency, as explained by Wardani et al. [34] in the context of fermented feed and high-quality concentrates. This aligns with the statement by Tüfekci and Olfaz [35] that the goal of fattening is to achieve the highest weight gain per head of livestock in the shortest possible time and at the lowest cost.

The results of this study indicate that the weight gain of goats at T0 and T1 was greater than at T2 and T3, or to increase weight by 1 kg, less feed was required (T0 and T1) (8.29 and 8.33 kg of DM) compared to T2 and T3, which required 9.06 and 10.45 kg of DM. The results of this study are also consistent with those reported by Tahuk and Bira [36], who found that Kacang goats fed a complete diet produced FCR values ranging from 8.56 ± 2.36 to 9.47 ± 2.85. The higher the quality of feed provided to livestock, the better the feed conversion efficiency produced, as it increases the rate of feed conversion in livestock. If the quality of feed improves, then increasing the DWG of livestock requires less feed compared to poor-quality feed.

These findings align with those of Mondal et al. [21], who reported that Black Bengal goats exhibited higher feed efficiency on the control diet compared to those fed diets containing 5% and 10% DRC. Similarly, Olafadehan et al. [22] observed that lambs fed diets containing 40% DRC achieved greater feed efficiency compared to those fed 0 and 20% DRC, but efficiency decreased significantly (*p* < 0.05) when the inclusion level was increased to 60%.

Taken together, these studies suggest that moderate inclusion levels of rumen content may improve feed efficiency due to better utilization of nutrient intake, whereas excessive inclusion can reduce efficiency, likely because of higher fiber content, lower energy density, and possible antinutritional factors in the material. The present results therefore support the notion that CRCS can be included up to 10% in goat diets without adverse effects on efficiency, but higher levels compromise nutrient utilization and growth performance.

### Economic efficiency of rations

Statistical test results show that the treatment of CRCS use in concentration significantly (*p* < 0.05) affects IOFC values. The higher the level of CRCS use, the higher the feeding cost per weight gain. Based on the data in Table 5, the highest IOFC values were observed in T1 and T0 [Indonesian rupiahs (IDR) 30,001 and 27,341], which were significantly higher than those in T2 and T3 (IDR 8,636 and 7,133). Although the use of CRCS at high levels (20% and 30%) resulted in a decrease in IOFC (from IDR 30,001 to IDR 7,133/h/day), it still contributed positively to environmental benefits by utilizing slaughterhouse waste. However, for smallholders, the economic loss may outweigh the ecological advantage unless further processing improves silage quality and animal performance.

**Table 5.** Economic value of goat feed in research.

**Parameters**	T0	T1	T2	T3	*p*-value
IOFC (IDR/head/day)	27,341ᵇ	30,001ᵇ	8,636ᵃ	7,133ᵃ	0.04
Feed cost per gain (IDR/kg DWG)	44.77 ± 9.43ᵃ	40.57 ± 3.33ᵃ	53.48 ± 11.54ᵇ	54.71 ± 11.66ᵇ	0.04

T0: Kume grass forage 70% + concentrate 30% (without CRCS)

T1: Kume grass forage 70% + concentrate 30% (concentrate contains 10% CRCS)

T2: Kume grass forage 70% + concentrate 30% (concentrate contains 20% CRCS)

T3: Kume grass forage 70% + concentrate 30% (concentrate contains 30% CRCS).

^a,b^Means on the same row with the same letter superscript are not statistically different (*p* < 0.05).

The FCPG value is also influenced by the use of CRCS in the concentrate (*p* < 0.05). The higher the level of CRCS use in the concentrate, the higher the feed cost per gain. In line with the IOFC parameter, the FCPG value in T1 (40.57 IDR/kg DWG) was lower than in T2 and T3 (53.48 and 54.71 IDR/kg DWG).

## Conclusion

These findings show that CRCS at 10% in concentrate optimizes feed efficiency and profitability while valorizing slaughterhouse waste, contributing to more sustainable goat production systems in resource-limited areas. These findings are valid under the conditions of this short term. Further research is needed to improve the quality of CRCS and assess its long-term effects on animal health and reproduction.

## List of abbreviations

BW, body weight; DMI, dry matter intake; DWG, daily weight gain; FCR, feed conversion ratio; FCPG, feed cost per gain; IOFC, income over feed cost; IDR, Indonesian rupiahs; LMO, local microorganism; DM, dry matter; OM, organic matter; CP, crude protein; CF, crude fiber; TDN, total digestible nutrient; GE, gross energy; NDF, neutral detergent fiber; ADF, acid detergent fiber; kg, kilogram; gm, gram; gm/h/day, grams per head per day; IDR/kg DWG, Indonesian rupiahs per kilogram daily weight gain; IDR/head/day, Indonesian rupiahs per head per day; Rp/head/day, rupiahs per head per day; %, percentage; kcal/kg, kilocalories per kilogram.
